# Stewarding the inappropriate diagnosis and treatment of urinary tract infection: leveraging the urinalysis to understand true antibiotic overuse

**DOI:** 10.1017/ash.2025.26

**Published:** 2025-02-17

**Authors:** Whitney Hartlage, Alyssa Y. Castillo, Zahra Kassamali Escobar, Maria Bajenov, Natalia Martinez-Paz, John B. Lynch, Chloe Bryson-Cahn, Jeannie D. Chan

**Affiliations:** 1 Veteran’s Affairs Salt Lake City Health Care System, Salt Lake City, UT, USA; 2 University of Washington Center for Stewardship in Medicine, Seattle, WA, USA; 3 Division of Infectious Diseases, University of Colorado, Aurora, CO, USA; 4 Fred Hutchinson Cancer Center, Seattle, WA, USA; 5 School of Pharmacy, University of Washington, Seattle, WA, USA; 6 Division of Allergy and Infectious Diseases, University of Washington School of Medicine, Seattle, WA, USA

## Abstract

We evaluated 249 asymptomatic patients receiving antibiotics for urinary infection: 222 had asymptomatic pyuria and/or nitrituria (ASPN) and 133 had asymptomatic bacteriuria (ASB, growth ≥10^5^ colony forming units/ml). ASPN identified 40% more cases of unnecessary antibiotics compared to ASB and may be a more comprehensive measure of unnecessary antibiotic use.

## Background

The appropriate diagnosis and treatment of urinary tract infection (UTI) is an important focus in antimicrobial stewardship programs (ASP).^1^ An accurate diagnosis requires *both* consistent genitourinary symptoms and bacteriuria, and antibiotics are recommended.^2,3^ In contrast, asymptomatic bacteriuria (ASB)—defined as bacterial growth ≥10^5^ colony forming units/ml without genitourinary symptoms—does not merit treatment outside the settings of pregnancy, some genitourinary surgeries, and the immediate post-kidney transplant period.^3^ Nevertheless, treatment of ASB remains common and represents an important source of antibiotic overuse.^3,4^

ASPs have historically focused on monitoring and reducing treatment of ASB as a strategy to curb inappropriate antibiotic use. However, using the definition of ASB to measure inappropriate antibiotic use has important shortcomings: (1) clinicians often make empiric treatment decisions based on urinalysis results alone, as urine culture data is rarely available during initial patient encounters (especially in rural or critical access hospitals (CAHs), where turnaround times are up to 7 days), and (2) the definition of ASB excludes patients without significant bacteriuria.^5^ We hypothesized that patients with a positive urinalysis and urine culture with low or no growth—who are notably excluded from the strict definition of ASB—may still receive antibiotics, and that measurements of antibiotic use for ASB alone may underrepresent the true prevalence of unnecessary antibiotic use for presumed UTI.

We characterize the overlap and differences in inappropriate antibiotic use among patients with ASB and asymptomatic patients with pyuria/nitrituria (hereafter referred to as “ASPN”). Our intent is to clarify if ASPN would provide a more comprehensive measurement of antibiotic overuse to better inform and optimize stewardship efforts.

## Methods

The University of Washington Center for Stewardship in Medicine (UW CSiM) is a collaboration between infectious diseases and ASP experts from UW Medicine and 87 community, rural, and CAHs. Ten CAHs enrolled in a cohort-based, intensive quality improvement program focused on UTI. Between September 8, 2022, and May 30, 2023, participating hospitals identified patients who underwent urine testing at their respective facility. Abstractors at each CAH retrospectively performed chart review and submitted de-identified data with patient demographics, clinical characteristics, laboratory and microbiology results, and antibiotics using a REDCap electronic data collection tool.^6^ UW CSiM faculty analyzed the results. The UW Institutional Review Board designated this study as quality improvement and not research.

Included patients were 18+ years and had urine testing performed (both a urinalysis and urine culture or urine culture alone) during an ambulatory, Emergency Department (ED), or inpatient encounter. Patients were excluded if they met ≥2 criteria for systemic inflammatory response syndrome (SIRS), received antibiotics for a concomitant infection, underwent urinalysis testing alone, or met criteria for treatment for asymptomatic bacteriuria (including pregnancy or upcoming genitourinary surgery). The presence of UTI was defined by urinary urgency or frequency, dysuria, costovertebral angle pain or tenderness, suprapubic pain, temperature > 38.0°C, or altered mental status plus a systemic sign of possible infection (leukocytosis >10,000 cells/mm^3^ and/or systolic blood pressure <90 mmHg,).^2,7^ Patients who underwent urine testing without signs or symptoms of UTI described above were categorized as “asymptomatic”. ASPN was defined as the presence of positive leukocyte esterase or WBC >10 or positive nitrites on urinalysis without signs or symptoms of UTI. ASB was defined as a urine culture with >100,000 colony forming units (CFU)/ml of 1 or more species of bacteria without any documented UTI symptoms, regardless of urinalysis results.

The primary outcome was the prevalence of inappropriate antibiotic treatment in asymptomatic patients with ASB and ASPN.

## Results

Ten CAHs submitted a total of 1,036 cases. We excluded 212 due to presence of ≥2 SIRS criteria (n = 164), concomitant bacterial infection (n = 29), age <18 (n = 11), and pregnancy (n = 8). Of 824 patients included, 347 (42%) lacked signs/symptoms of UTI, 282 (34%) had ASPN, and 153 (19%) had ASB (Table 1).

**Table 1.** Baseline and clinical characteristics

	Asymptomatic for UTI, N = 347		Pyuria/Nitrituria (ASPN), N = 282		ASB with >100,000 CFU/ml, N = 153	
Variables	Treated n = 249 (%)	Not Treated n = 98 (%)	Treated, n = 222 (%)	Not Treated, n = 60 (%)	Treated, n = 133 (%)	Not Treated, n = 20 (%)
Age, median y [IQR]	76 [66–84]	71 [53–80]	76 [66–84]	72 [51–82]	76 [66–82]	77 [70–87]
Sex, female	174 (70)	64 (65)	154 (69)	45 (75)	91 (68)	17 (85)
Chronic catheter use	27 (11)	4 (4)	25 (11)	2 (3)	17 (13)	2 (10)
Location of urine study collection						
ED	193 (78)	76 (78)	177 (80)	45 (75)	101 (76)	15 (75)
Ambulatory care	43 (17)	14 (14)	34 (15)	11 (18)	24 (18)	2 (10)
Inpatient	13 (5)	8 (8)	11 (5)	4 (7)	8 (6)	3 (15)
Altered mental status alone	36 (14)	10 (10)	34 (15)	7 (12)	22 (17)	3 (15)
Positive urinalysis result						
Leukocyte esterase	202 (81)	53 (54)	202 (91)	53 (88)	110 (83)	12 (60)
WBC >10	148 (59)	24 (24)	148 (67)	24 (40)	100 (75)	12 (60)
Nitrites	93 (37)	10 (10)	93 (42)	10 (17)	91 (68)	6 (30)
Urine culture with no growth	39 (16)	44 (45)	32 (14)	23 (38)	–	–
Urine culture with any quantity of growth	210 (84)	54 (55)	190 (86)	37 (62)	–	–
>100,000 CFU/ml	147 (59)	22 (22)	137 (72)	16 (43)	133 (100)	22 (100)
51,000–100,000 CFU/ml	35 (14)	8 (8)	29 (21)	5 (14)	–	–
11,000–50,000 CFU/ml	19 (8)	18 (18)	15 (8)	13 (35)	–	–
≤10,000 CFU/ml	9 (4)	6 (6)	9 (5)	3 (8)	–	–

Abbreviations: UTI, Urinary tract infection; ASPN, asymptomatic pyuria and/or nitrituria; ASB, asymptomatic bacteriuria; IQR, Interquartile range; ED, Emergency Department; WBC, white blood cell count; CFU, Colony forming unit.

**Figure 1.**
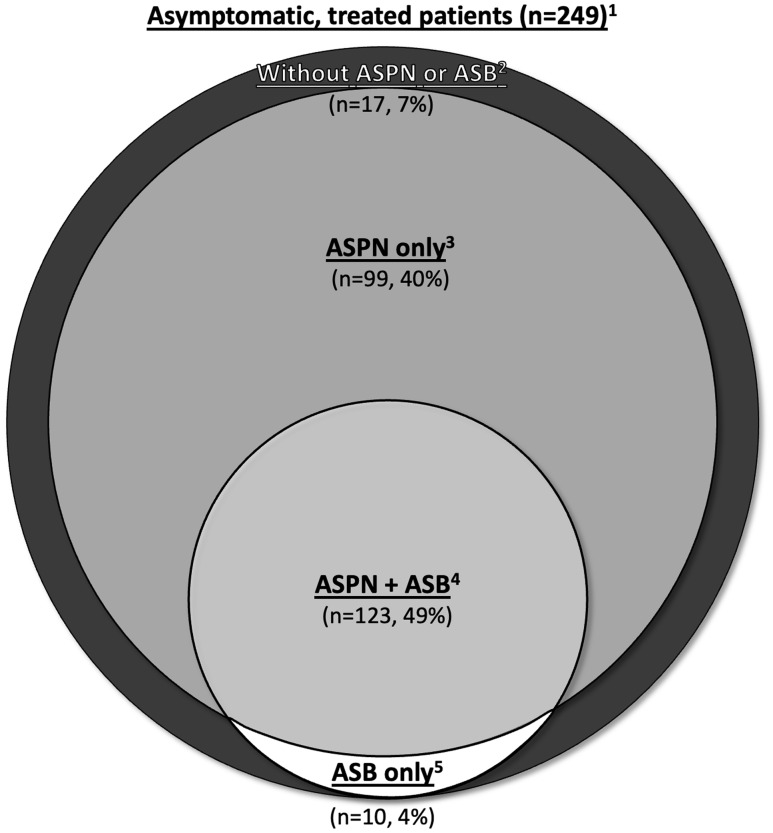
Measurements of inappropriate antibiotic use for suspected urinary tract infection (UTI) using various study definitions. ^1^Asymptomatic, treated patients: the overall number of patients asymptomatic for UTI that underwent urine testing and received antibiotic treatment. ^2^Without asymptomatic pyuria/nitrituria (ASPN) or asymptomatic bacteriuria (ASB): among the asymptomatic, treated patients, the proportion without ASPN or ASB. ^3^ASPN: prevalence of ASPN among asymptomatic, treated patients. ^4^ASPN + ASB: prevalence of ASPN AND ASB among asymptomatic, treated patients. ^5^ASB: prevalence of ASB among asymptomatic, treated patients.

Among 347 asymptomatic patients, 249 (72%) received antibiotics. Among the 249 treated patients, 222 (89%) had ASPN, 133 (53%) had ASB, and 123 (49%) had both ASPN and ASB. ASPN criteria identified 99 (40%) additional cases of unnecessary antibiotic use compared to ASB (Figure 1). Ten cases (4%) had ASB and not ASPN.

## Discussion

Our analysis demonstrated that ASPN captured a larger proportion of patients inappropriately treated with antibiotics (89%) than the traditional ASB definition (53%). While there was significant overlap between the groups, an additional 99 (40%) patients met criteria for ASPN but *not* ASB. This suggests the current guideline classification of ASB incompletely captures antibiotic prescribing and that ASPN may be a broader measure to monitor and target unnecessary antibiotic use for UTI.

Although our study does not identify the underlying causes for why ASPN is a more comprehensive measure of inappropriate antibiotic use, we postulate several possible causes. Many clinicians may have an incomplete understanding of the urinalysis test characteristics (low positive predictive value),^8,9^ and may interpret an abnormal urinalysis as “diagnostic” of a UTI; therefore, pyuria or nitrituria may be a strong impetus to initiate antibiotics. In a retrospective cohort of asymptomatic patients with urinalysis performed within a preoperative screening protocol, the presence of pyuria increased the odds of antibiotic prescribing 4-fold.^9^ Secondly, hospital microbiology labs may not define an “abnormal” urine culture strictly as >100,000 CFU/ml of a uropathogen; if less stringent criteria are used, results of low colony count or mixed flora may still be flagged as abnormal, prompting treatment but not meeting a strict ASB definition.

The distinction between ASB and ASPN has little clinical relevance, as asymptomatic patients (regardless of urinalysis or culture results) do not merit antimicrobial treatment. However, this distinction is highly consequential from a surveillance perspective for ASPs aiming to accurately measure inappropriate antibiotic use for UTI and implement impactful interventions to reduce it. We propose that ASPs could maximize their impact by including abnormal urinalysis to prompt case review (rather than only those with positive urine cultures)—which would in turn enable stewards to provide audit and feedback to clinicians who inappropriately treat ASPN, capturing a larger proportion of inappropriate antibiotic use. Careful review of ASPN cases may yield additional insights, including the need for diagnostic stewardship interventions to reduce inappropriate urinalysis collection and clinician education on urinalysis interpretation.

Our study has several limitations. Data were obtained via retrospective chart review and dependent upon accurate documentation of symptoms and exam by the evaluating provider. Second, it is possible that urinalyses may influence prescribing more in CAHs with resource constraints and a longer turn-around-time for urine culture results, limiting generalizability to larger urban hospitals. Lastly, all patients had both a urinalysis and urine culture obtained during their clinical encounter (95%) or a urine culture obtained alone (5%); individuals who underwent urinalysis testing alone (and subsequent empiric treatment) were not included, and our study may underestimate the true rate of ASPN antibiotic prescribing.

In conclusion, a urinalysis with pyuria and/or nitrituria in patients without urinary symptoms is associated with high rates of antibiotic treatment in CAHs. Our study suggests that including patients with ASPN would provide a more comprehensive means of quantifying unnecessary antibiotic prescribing and may better inform ASP efforts. Additional studies are needed to identify the prevalence of ASPN in other settings and measure the impact of stewardship program review and intervention.
